# The influence of maternal pregnancy glucose concentrations on associations between a fetal imprinted gene allele score and offspring size at birth

**DOI:** 10.1186/s13104-018-3933-1

**Published:** 2018-11-19

**Authors:** Clive J. Petry, Ken K. Ong, Ieuan A. Hughes, Carlo L. Acerini, David B. Dunger

**Affiliations:** 1 Department of Paediatrics, Addenbrooke’s Hospital, University of Cambridge, Hills Road, Box 116, Cambridge, CB2 0QQ UK; 2 Medical Research Council Epidemiology Unit, University of Cambridge, Cambridge, CB2 0QQ UK; 3 The Institute of Metabolic Science, University of Cambridge, Cambridge, CB2 0QQ UK

**Keywords:** Gestational diabetes, Pregnancy, Growth, Development, Cohort study

## Abstract

**Objective:**

Previously we found that certain fetal imprinted genes represented as an allele score are associated with maternal pregnancy glucose concentrations. Recently it was reported that fetal polymorphisms with strong associations with birth weight tend to mediate these independently of increases in maternal pregnancy glucose concentrations. We therefore investigated whether potential associations between the fetal allele score and birth weight were related to maternal glucose concentrations in the Cambridge Baby Growth Study.

**Results:**

The fetal imprinted gene allele score was positively associated with birth weight (β = 63 (17–109) g/risk allele, β′ = 0.113, p = 7.6 × 10^−3^, n = 405). This association was partially attenuated by adjusting for maternal glucose concentrations (β = 50 (4–95) g/risk allele, β′ = 0.089, p = 0.03, n = 405). The allele score was also positively associated with risk of being large for gestational age at birth (odds ratio 1.60 (1.19–2.15) per risk allele, p = 2.1 × 10^−3^, n = 660) and negatively associated with risk of being small for gestational age at birth (odds ratio 0.65 (0.44–0.96) per risk allele, p = 0.03, n = 660). The large for gestational age at birth association was also partially attenuated by maternal glucose concentrations. These results suggest that associations between the fetal imprinted gene allele score and size at birth are mediated through both glucose-dependent and glucose-independent mechanisms.

## Introduction

Fetal exposure to glucose is thought to be one of the principal stimulators of growth in utero [1–3]. It is believed that glucose-stimulated fetal insulin secretion stimulates growth whether or not pregnancies are affected by diabetes [4]. Indeed particularly prior to the third trimester of pregnancy when its glucoregulatory function develops, the principal roles of fetal insulin are both mitogenic and anabolic, such as enhancing the growth of white adipocytes and stimulating triglyceride production and deposition in them [5].

Genetics can have roles in regulating both maternal glucose concentrations in pregnancy and fetal growth expressed as offspring birth weight. We recently reported associations between fetal imprinted genes represented as an allele score and both gestational diabetes and maternal glucose concentrations in late pregnancy [6]. In a birth weight-related genome wide association study (GWAS) Beaumont et al. [7] recently reported certain maternal polymorphic variants that were associated with offspring birth weights where the equivalent fetal variants were not. In contrast certain other variants were associated with offspring birth weight in both the maternal and fetal genes. Interestingly all these maternal variants, where the fetal equivalent were not associated with offspring birth weights, were also associated with maternal glucose concentrations in pregnancy [7]. A subsequent study found that a gene score constructed using the principal fetal birth weight variants (from [8]) was strongly associated with birth weights independently of effects of maternal glucose concentrations [9]. The authors of this study concluded that for any level of maternal glucose concentration fetal genetics has a major impact on growth but acts predominantly through mechanisms independent of maternal glucose. However none of the fetal variants used to construct the gene score in this study were independently associated with maternal glucose concentrations. In the current study we therefore examined relationships between our fetal imprinted gene allele score and measures of size at birth, in particular investigating whether any allele score associations with size at birth appear to be attenuated by maternal glucose concentrations in pregnancy.

## Main text

### Methods

#### Cambridge baby growth study

The first phase of the prospective, longitudinal Cambridge Baby Growth Study recruited mothers (and their partners and offspring) attending early pregnancy ultrasound clinics at the Rosie Maternity Hospital, Cambridge, UK between the years 2001–2009 [6, 10]. At around 28 weeks of gestation the mothers underwent a 75 g oral glucose tolerance test (OGTT) after fasting overnight. Plasma glucose concentrations were measured using a standard glucose oxidase-based procedure on samples collected when fasting and 60 min after the consumption of the glucose load. Offspring birth weight and gestational age at birth data were collected from hospital notes. Large for gestational age (LGA) at birth was defined as being in the top decile for birth weight adjusted for gestational age. Similarly small for gestational age at birth (SGA) was defined as being in the bottom decile. In this cohort 96.9% of the offspring were Caucasian, 0.8% were mixed race, 0.6% were Afro-Caribbean, 0.8% were Oriental and 0.9% were Indo-Asian. The current analysis was restricted to pregnancies where both fasting and 60 min OGTT glucose concentrations were available.

#### Genotyping and fetal allele score formulation

Blood or mouth swab samples for DNA extraction were collected from 845 family (mother, father and baby) trios of the 1074 families where maternal OGTT data were available. Genomic DNA was extracted from these samples using an Autopure LS Machine (Qiagen Ltd., Crawley, UK). Allelic transmission to the fetus was imputed from the DNA family trio genotypes [6, 10], with the genotyping performed using Kompetitive Allele Specific PCR assays (by LGC Genomics, Hoddesdon, UK). The genotypes that were used in this study were all consistent with Hardy–Weinberg equilibrium (p > 0.05 using the χ^2^ test) and had a repeat genotyping discordancy rate of < 1.0%. The unweighted fetal allele score was formulated as previously described [6] using the fetal paternally-transmitted *INS*-*IGF2* rs10770125 and rs2585, and maternally-transmitted *KCNQ1* rs231841 and rs7929804 alleles.

#### Statistical analysis

Associations with offspring birth weight were tested using linear regression, adjusted for established co-variates. Associations with LGA and SGA were tested using both logistic and linear regression. P < 0.05 was considered statistically significant throughout. Data are mean (95% confidence interval) unless stated otherwise.

### Results

#### Associations with offspring birth weight

**Fig. 1:**
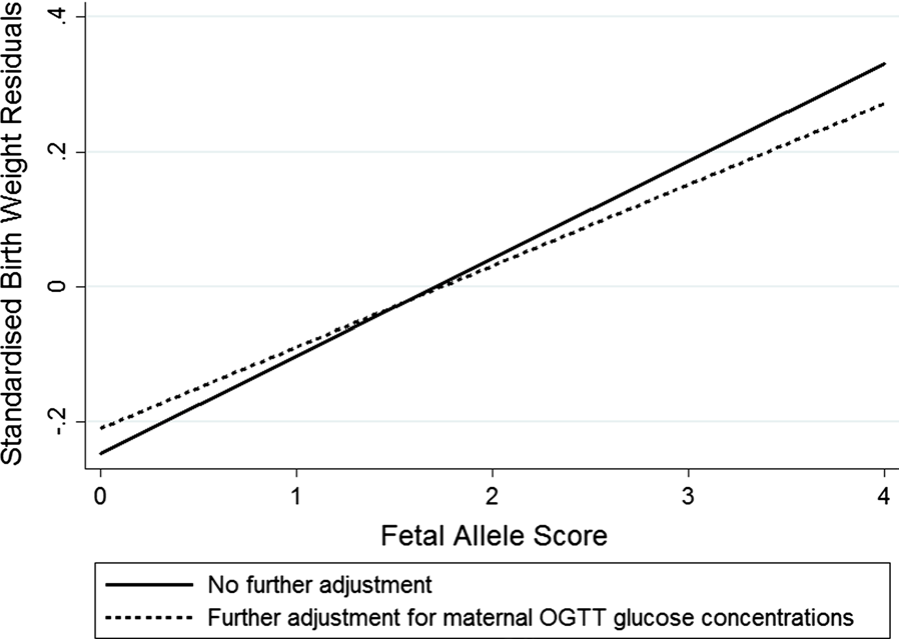
Linear fit prediction plots showing the relationships between the fetal allele score and standardised birth weight residuals (after adjustment for gestational age at birth, sex, parity, maternal pre-pregnancy body mass index, pregnancy weight gain and maternal smoking during pregnancy). The plots are shown with and without further adjustment for maternal OGTT glucose concentrations, this adjustment flattening the slope

The covariates in the regression models (gestational age at birth, sex, parity, maternal pre-pregnancy body mass index, pregnancy weight gain and maternal smoking during pregnancy) explained 31.5% of the variance in the offspring birth weights by themselves. OGTT fasting glucose concentrations were significantly associated with offspring birth weights when added to the model (β = 0.16 (0.09–0.23) g l/mmol, β′ = 0.150, p = 1.5 × 10^−5^, n = 609), as were OGTT 60 min glucose concentrations (β = 0.05 (0.03, 0.07) g l/mmol, β′ = 0.161, p = 4.4 × 10^−6^, n = 602). The fetal allele score was also positively associated with birth weight (β = 63 (17–109) g/risk allele, β′ = 0.113, p = 7.6 × 10^−3^, n = 405) (Fig. 1). This association was partially attenuated when adjusting for the OGTT fasting and 60 min glucose concentrations (β = 50 (4–95) g/risk allele, β′ = 0.089, p = 0.03, n = 405), shown by the flatter slope of the predicted line of best fit of the model (Fig. 1).

#### Associations with being large or small for gestational age at birth

**Fig. 2:**
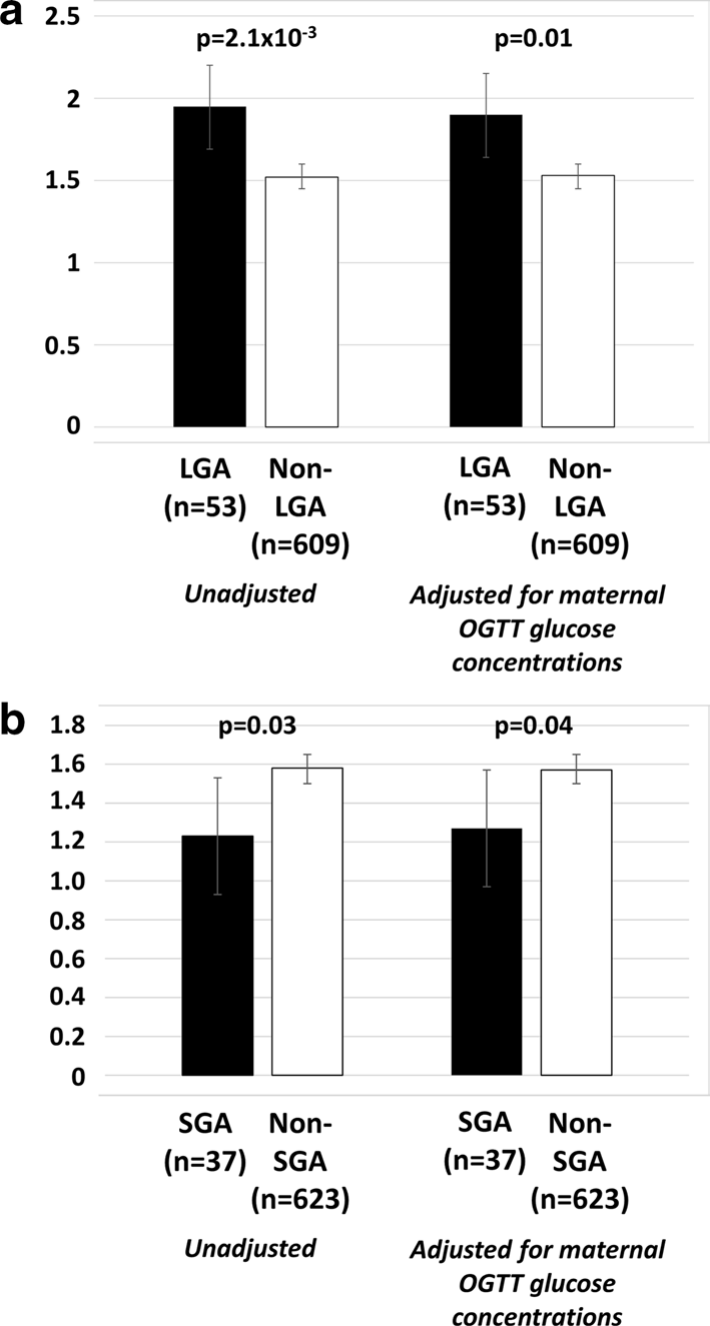
The fetal allele score in those born (**a**) LGA or (**b**) SGA and those not born LGA/SGA, with and without adjustment for maternal OGTT glucose concentrations. Data are means (95% confidence intervals)

The fetal allele score was positively associated with risk of being LGA [odds ratio (OR) 1.60 (1.19–2.15) per risk allele, p = 2.1 × 10^−3^] (Fig. 2a). The association was only partially attenuated by adjusting for OGTT fasting and 60 min glucose concentrations [OR 1.47 (1.09–1.98) per risk allele, p = 0.01)]. The fetal allele score was also negatively associated with risk of being SGA [OR 0.65 (0.44–0.96) per risk allele, p = 0.03] (Fig. 2b). The association was not attenuated when adjusting for OGTT fasting and 60 min glucose concentrations [OR 0.67 (0.49–0.99) per risk allele, p = 0.04].

### Discussion

In this study the fetal imprinted gene allele score that we had previously shown to be associated with both maternal glucose concentrations and gestational diabetes risk [6], was additionally associated with offspring birth weight and risk of being LGA or SGA. This is perhaps not surprising given the enrichment of imprinted gene regions identified in the largest birth weight-related GWAS using fetal genotypes [8]. The effect sizes were partially attenuated when the associations were further adjusted for week 28 OGTT fasting and 60 min maternal glucose concentrations, suggesting that the link between the fetal allele score and birth weight is mediated through both glucose-dependent and glucose independent mechanisms. These findings are therefore somewhat inconsistent with the associations reported by Hughes et al. [9] where their fetal gene score was associated with birth weight completely independently of maternal glucose concentrations. The difference in the results of the two studies is probably due to the way that the two fetal gene scores were formulated: that used by Hughes et al. [9] being put together from fetal polymorphisms strongly associated with birth weights and our allele score being established using fetal alleles found to be associated with maternal glucose concentrations. Whilst increased maternal glucose concentrations are known to lead to increased birth weights [4], glucose-independent pathways upregulated in gestational diabetes that could potentially affect offspring birth weight include increased placental transport of both fatty acids and certain amino acids [11, 12].

The main strengths of this study are its prospective nature and the use of a novel fetal imprinted gene allele score that we found to be robustly associated with maternal glucose concentrations, even to genome wide significance levels by meta-analysis of three different birth cohorts [6]. Its conclusion is that some of the principal fetal imprinted gene variants that are associated with maternal glucose concentrations in late pregnancy in our studies (fetal paternally-transmitted *INS*-*IGF2* rs10770125 and rs2585, and maternally-transmitted *KCNQ1* rs231841 and rs7929804 [6, 10]), are collectively associated with birth weight through both maternal glucose-dependent and glucose-independent mechanisms.

## Limitations

- The study has a modest (and variable) sample size which restricted its statistical power.
- The lack of validation of the associations in additional cohorts.
- The lack of adjustment of the p-values for multiple testing.

## Abbreviations

GWAS: genome wide association study
LGA: large for gestational age
OGTT: oral glucose tolerance test
OR: odds ratio
SGA: small for gestational age
